# Role of Estrogen Response Element in the Human Prolactin Gene: Transcriptional Response and Timing

**DOI:** 10.1210/me.2015-1186

**Published:** 2015-12-21

**Authors:** Anne V. McNamara, Antony D. Adamson, Lee S. S. Dunham, Sabrina Semprini, David G. Spiller, Alan S. McNeilly, John J. Mullins, Julian R. E. Davis, Michael R. H. White

**Affiliations:** Systems Microscopy Centre (A.V.M., A.D.A., D.G.S., M.R.H.W.), Faculty of Life Sciences, and Faculty of Medical and Human Sciences (L.S.S.D., J.R.E.D.), Centre for Endocrinology and Diabetes, Institute of Human Development, University of Manchester, Manchester M13 9PT, United Kingdom; and The Molecular Physiology Group (S.S., J.J.M.), Centre for Cardiovascular Science, and Medical Research Council Human Reproductive Sciences Unit (A.S.M.), Centre for Reproductive Biology, Queen's Medical Research Institute, Edinburgh EH16 4TJ, United Kingdom

## Abstract

The use of bacterial artificial chromosome (BAC) reporter constructs in molecular physiology enables the inclusion of large sections of flanking DNA, likely to contain regulatory elements and enhancers regions that contribute to the transcriptional output of a gene. Using BAC recombineering, we have manipulated a 160-kb human prolactin luciferase (hPRL-Luc) BAC construct and mutated the previously defined proximal estrogen response element (ERE) located −1189 bp relative to the transcription start site, to assess its involvement in the estrogen responsiveness of the entire hPRL locus. We found that GH3 cell lines stably expressing Luc under control of the ERE-mutated hPRL promoter (ERE-Mut) displayed a dramatically reduced transcriptional response to 17β-estradiol (E2) treatment compared with cells expressing Luc from the wild-type (WT) ERE hPRL-Luc promoter (ERE-WT). The −1189 ERE controls not only the response to E2 treatment but also the acute transcriptional response to TNFα, which was abolished in ERE-Mut cells. ERE-WT cells displayed a biphasic transcriptional response after TNFα treatment, the acute phase of which was blocked after treatment with the estrogen receptor antagonist 4-hydroxy-tamoxifen. Unexpectedly, we show the oscillatory characteristics of hPRL promoter activity in individual living cells were unaffected by disruption of this crucial response element, real-time bioluminescence imaging showed that transcription cycles were maintained, with similar cycle lengths, in ERE-WT and ERE-Mut cells. These data suggest the −1189 ERE is the dominant response element involved in the hPRL transcriptional response to both E2 and TNFα and, crucially, that cycles of hPRL promoter activity are independent of estrogen receptor binding.

Prolactin (PRL) is a polypeptide hormone primarily produced by the lactotroph cells of the anterior pituitary. It is also expressed in humans and primates at extrapituitary sites, including the endometrium and in immune tissues (1–4), and has been reported to have a wide range of biological actions (5). PRL expression and secretion from lactotroph cells is subject to both acute and long-term regulation by multiple hormone signals, including hypothalamic TRH, dopamine, and second messengers Ca^2+^ and cAMP (6).

Estrogen is a well-known stimulus to pituitary PRL gene expression in rodents and in man (7–11). Estrogen-induced gene expression is mediated by the actions of the 2 estrogen receptors (ERs), ERα and ERβ. These ligand-activated transcription factors regulate gene expression through direct binding to estrogen response elements (EREs) in the DNA of target promoters or indirectly by binding to DNA-bound transcription factors such as activating protein 1 (AP-1) and specificity protein 1 (Sp1). Once bound, the ER then facilitates recruitment of coregulator proteins to the promoter to influence transcriptional activity (12–15). ER signaling can also be activated in the absence of ligand by a growing number of growth factors and cytokines, including epidermal growth factor, fibroblast growth factor 2 (FGF-2), and TNFα, which trigger signal transduction pathways such as ERK and MAPK that can phosphorylate and activate ER in the absence of ligand (16–18). Additionally, targeted deletion of ERα (ERαKO) in mice leads to reduced lactotroph cell numbers and a dramatic reduction in pituitary PRL mRNA (19).

Cyclical recruitment of ER to the promoter regions of estrogen-responsive genes has been reported in several systems (12, 17, 20). Furthermore, ER-binding sites can be situated at long-range to the transcription start site of the genes they regulate. Binding of ER to distal response elements promotes similar behavior to that at proximal elements, with recruitment of coregulators to influence chromatin structure and transcriptional activity. Long-range chromatin interactions between ERs bound at distal response elements and those located in the proximal regions of estrogen-regulated genes has also been reported (21–23).

In the human PRL (hPRL) gene promoter we have previously characterized a variant ERE 1189 bp upstream from the transcription start site which confers modest transcriptional induction on reporter gene expression after estrogen stimulation in cultured GH3 pituitary cells expressing luciferase (Luc) under the control of a 5-kb hPRL promoter fragment (9). In contrast, GH3 cells stably transfected with a bacterial artificial chromosome (BAC) construct expressing Luc under the control of a 160-kb fragment of the hPRL gene locus display a greater transcriptional response to estrogen than the 5-kb promoter construct (1). Either the known −1189 ERE is a crucial response element and full transcriptional activation in response to 17β-estradiol (E2) depends on genomic context and other enhancer elements, or there are additional upstream EREs that contribute to the hPRL E2 response. To test this hypothesis we have used BAC-recombineering to mutate 4 base pairs of the −1189 ERE in the hPRL BAC construct (hPRL ERE-Mut BAC). Here, we report that GH3 cells stably transfected with this construct have significantly reduced estrogen sensitivity compared with those stably transfected with the hPRL BAC, suggesting that even in the large 160-kb genomic fragment, this proximal ERE is the dominant response element involved after E2 treatment. Additionally, we show, using real-time bioluminescence imaging of hPRL ERE-Mut directed Luc activity in single transfectant cells that pulsatile cycles of hPRL transcription persist and are therefore not dependent on ER binding to the proximal promoter region.

## Materials and Methods

### Transgene mutation

The PRL-Luc reporter BAC has been previously described (1) In order to mutate the previously defined ERE (9), the hPRL-Luc BAC was maxiprepped using Macherey-Nagal Nucleobond BAC100 kit (Thermo Fisher) and 100 ng used to transform SW102 *Escherichia coli* cells by electroporation (1.8 kV, 200 Ω, 25 mF). Clones were selected, miniprepped, and assayed by pulsed field gel electrophoresis with *Sal*I and *Not*I digestions to isolate a SW102-Prl-Luc positive clone. Seamless recombination to mutate the ERE was achieved using methods described by (24). Briefly, chimeric PCR was used to amplify a homology arm tagged GalK expression cassette (H-GalK-H) and used in primary recombination (positive clones selected on galactose containing minimal media) to replace the wild-type (WT) ERE sequence. A second round of recombination with the same homology arms flanking a mutated ERE sequence amplified from vector phPrl5000mutERE (9) was performed (positive clones selected on deoxygalactose and glycerol containing minimal media). Clones were screened by amplification of the ERE region and subsequent *Eoc*NI digest of amplicon (the mutation results in the formation of this restriction site). BAC DNA was also digested with appropriate restriction enzymes and pulsed field gel electrophoresis confirmed overall BAC size and structure was maintained.

Primers were as following: chimeric EREmutGalK-F (GalK-specific sequence in bold) ATTCATTATAGTCATTTCATTTAGGAAATTTCCAAAAGGTGAATGGAATTTTTAAGCCCATGAAAGATGAATTTT**CCTGTTGACAATTAATCATCGGCA**, chimeric EREmutGalK-R CAAGGATAGCAGTGTGTAAACTGTTATTAACTTATGATCTCTCTACCTTCTCTGACCCTGAGCCACTCTGAGGCC**TCAGCACTGTCCTGCTCCTT**, hPrlmutERE-F AGGCTGCTTTAGATGCATGG, and hPrlmutERE-R GTTTTCCAGGGCAAACACAC.

### Cell culture and generation of stable transfectant BAC cell lines

Cells were maintained in phenol red-free DMEM with pyruvate (Gibco) and supplemented with 1% glutamine and 10% fetal bovine serum (Gibco). Serum starving conditions (hormone free) for 24 hours were phenol red-free DMEM with pyruvate (11880; Gibco) supplemented with 1% glutamine and 0.25% BSA. With the exception of FGF-2 (Merck Biosciences) and E2 (Abcam), all reagents were obtained from Sigma.

For the generation of the ERE-WT and ERE-Mut hPRL-Luc BAC cell lines, 5 × 10^6^ GH3 cells were seeded into 10-cm dishes and left to adhere overnight. BAC DNA was prepared by maxiprep (BAC100 Nucleobond kit; Macherey-Nagel), and 3 μg were used to transfect 5 × 10^6^ cells in a 10-cm dish using ExGen500 transfection reagent. Media were changed 3 days after transfection and supplemented with 500-μg/mL G418. Media and antibiotic were refreshed every 3–4 days. Colonies formed 2–3 weeks after culturing in selection media were ring cloned into individual wells of a 48-well plate and sequentially scaled up to large culture vessels as necessary. The clones were screened for Luc expression and responses to stimuli (10nM E2, 5μM forskolin [FSK], 10-ng/mL FGF-2, 10nM dexamethasone [Dex], and 10-ng/mL TNFα).

### Endpoint luminometry assays

1 × 10^5^ GH3 cells per well were seeded into 24-well plates, serum starved for 24 hours, then stimulated in triplicate as indicated. After treatment medium was removed, cells washed once with PBS, then lysed with 200 μL of lysis buffer per well (25mM Tris/PO_4_, 10mM MgCl_2_, 5mM EDTA, 15% glycerol, 0.1% Triton X-100, and 0.1-mg/mL BSA). Cell lysis was aided by agitation at room temperature for 15 minutes, ATP added to a final concentration of 1mM, and Luc activity of samples measured using a FLUOstar Omega (BMG Labtech). Experiments were performed in triplicate at least 3 times. Results are shown as mean fold induction relative to an unstimulated control ± SD of at least 3 independent experiments. For statistical analysis, *P* values were calculated using Student's *t* test.

### Live-cell luminometry assays

1.5 × 10^4^ GH3 cells per well were seeded into white opaque 96-well microplates (PerkinElmer), serum starved for 24 hours in media containing 1mM luciferin, then stimulated in triplicate as indicated. Microplates were covered with Breathe-Easy sealing membranes (Sigma) and Luc activity of wells measured using a FLUOstar Omega (BMG Labtech) over a 24-hour period with cells maintained at 37°C with 5% CO_2_-95% air. Photon counts for each well were integrated over 5 seconds and repeated every 15 minutes. Results are shown as mean fold induction relative to an unstimulated control ± SD of at least 3 independent experiments. For statistical analysis, *P* values were calculated using Student's *t* test.

### Real-time luminescence imaging

1.2 × 10^5^ GH3 cells were seeded in 35-mm glass coverslip-based dishes (Greiner), cultured in media containing 1mM luciferin. Luciferin was added at least 12 hours before the start of the experiment. Cells treated with 10-ng/mL TNFα or 10nM E2 were maintained in hormone-free media for 24 hours before stimulation. Cells were transferred to the stage of a Zeiss Axiovert 200 equipped with an XL incubator (maintained at 37°C in a 5% CO_2_-95% air in humidified conditions) in a dark room. Luminescence images were collected using a Fluar 10×, 0.5-NA objective (Zeiss), and captured using a photon-counting charge coupled device camera (Orca II BT; Hamamatsu Photonics). Sequential images were taken with a 10-minute integration period, then analyzed using Kinetic imaging software AQM6 (Andor).

### Stochastic switch model (SSM) analysis

Raw luminescence data was analyzed using a SSM to estimate the probability of switches between variable transcription rates (described in Ref. 26). This highly iterative reverse jump Markov chain Monte Carlo algorithm back-calculates from observed Luc activity using known protein and mRNA degradation rates producing a probability distribution of transcription phase durations. Matlab 2014a software (MathWorks), including the Bioinformatics and Statistical toolboxes were used for mathematical analyses.

## Results

### Mutation of the −1189 ERE in the hPRL BAC-Luc construct (ERE-Mut BAC-Luc)

**Figure 1.**
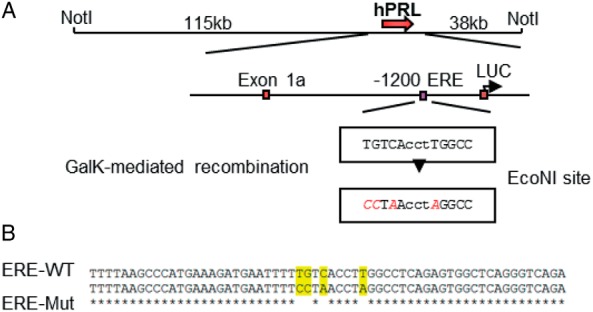
Mutation of the −1189 ERE in the hPRL BAC-Luc construct (ERE-Mut BAC). Mutagenesis schematic of the −1189 ERE mutation in the hPRL-Luc BAC construct. GalK-mediated recombination was used to mutate 4 base pairs in the ERE, introducing an *Eco*NI site (A). Sequencing confirmation of the 4 base pair mutations in the −1189 ERE, ERE-WT top line, ERE-Mut bottom line (B).

The hPRL-Luc reporter BAC (hPRL BAC-Luc) has been described previously. This reporter construct contains approximately 115 kb of 5′-flanking sequence from the hPRL genomic locus, including the previously characterized ERE at −1189 bp (1). Using BAC recombineering we have mutated 4 conserved base pairs of the −1189 ERE in the hPRL BAC-Luc construct (Figure 1, A and B, and Supplemental Figure 1, A–C) to disrupt the sequence and ER binding. Stably transfected pituitary GH3 clonal cell lines expressing either hPRL ERE-Mut BAC-Luc (here termed ERE-Mut) or hPRL WT-ERE BAC-Luc (here termed ERE-WT) were then generated and clonal lines screened for basal Luc activity to select for positive transfectant clones.

### Mutation of the −1189 ERE significantly reduces estradiol-mediated activation of hPRL transcription

**Figure 2.**
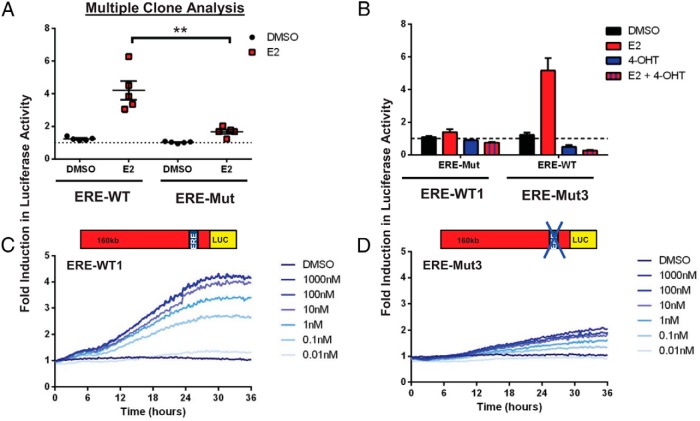
Estrogen responsiveness of ERE-WT and ERE-Mut BAC cell lines. E2 stimulation (10nM) of 5 individual serum-starved (24 h) ERE-WT and ERE-Mut clones; after treatment for 24 hours with either 10nM E2 or vehicle control (0.1% dimethylsulfoxide [DMSO]), cells were lysed and assayed for Luc activity; **, *P* < .001 (A). Effect of E2 cotreatment with the E2 antagonist 4-OHT (1000nM) in ERE-WT1 and ERE-Mut3 cell lines (B). Live cell luminometry of serum-starved (24 h) ERE-WT1 (C) and ERE-Mut3 (D) cell lines using increasing concentrations of E2 with Luc activity measured every 15 minutes. The dotted line indicates the baseline determined from untreated control samples. Results are shown as fold induction ± SD from the untreated control sample with values representing the mean of at least 3 independent experiments, each with 3 replicates.

The estrogen responsiveness of 5 ERE-Mut and 5 ERE-WT clones were compared. Cells were maintained in hormone-free medium for 24 hours, treated with 10nM E2 (Figure 2A), then lysed and Luc activity measured. All 5 ERE-WT and 3 of the ERE-Mut clones showed a significant induction in reporter gene activity after E2 treatment. However, ERE-Mut clones all showed significantly reduced transcriptional activity compared with their ERE-WT counterparts, with a mean fold induction of 1.6 compared with a 4.2-fold mean induction, respectively. Cell proliferation assays showed a small but nonsignificant increase in cell number over the 24-hour period after E2 treatment (data not shown). Estrogen activation of the endogenous rat PRL (rPRL) promoter has previously been shown in both the GH3 cell line and primary cultures of Fischer-344 rat pituitary glands, with a 4-fold activation observed in both using quantitative PCR after a 24-hour exposure to 10nM E2 (9).

Additional experiments were then carried out on 2 selected GH3 stable transfectant clonal lines, ERE-WT1 and ERE-Mut3. Cotreatment of E2 with the anti-estrogen 4-hydroxy-tamoxifen (4-OHT) abolished E2-mediated reporter gene expression in both ERE-WT and ERE-Mut cell lines, suggesting ER involvement in the inductions seen (Figure 2B). Real-time live-cell luminometry of cell populations after treatment with increasing concentrations of E2 (0.01nM–1000nM) over time showed a progressive, dose-dependent increase in reporter gene expression in both cell lines but with significantly reduced transcriptional activity observed in ERE-Mut3 compared with ERE-WT1 (Figure 2, C and D).

### Pulsatile cycles of hPRL gene transcription are independent of ER binding

**Figure 3.**
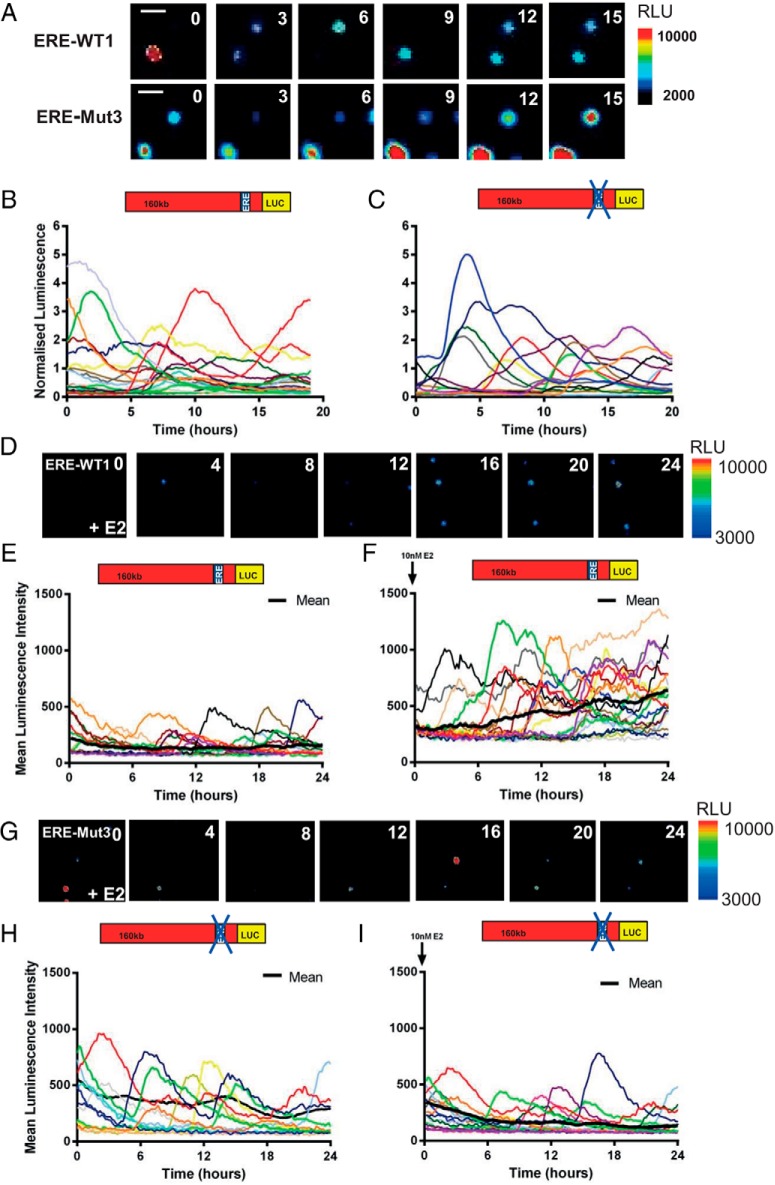
Single cell transcriptional activity in ERE-WT and ERE-Mut BAC cell lines. Luminescence signal from ERE-WT1 and ERE-Mut3 BAC cell lines in serum containing media. Representative images of ERE-WT1 and ERE-Mut3 BAC cells, white line represents scale bar equal to 50μM (A). Single cell transcriptional traces from unstimulated ERE-WT1 (B) and ERE-Mut3 BAC cells (C) in serum containing media. Luminescence signal from serum-starved ERE-WT1 cells: representative images after 10nM E2 treatment (D) and single cell transcriptional traces from untreated (E) and 10nM E2-treated cells (F). Luminescence signal from serum-starved ERE-Mut3 cells. Representative images after 10nM E2 treatment (G) and single cell transcriptional traces from untreated (H) and 10nM E2-treated cells (I). Colored lines represent data from single cells with the thick black line representing the mean population response; n = 21 cells (E), n = 19 cells (F), n = 22 cells (H), and n = 18 cells (I).

**Figure 4.**
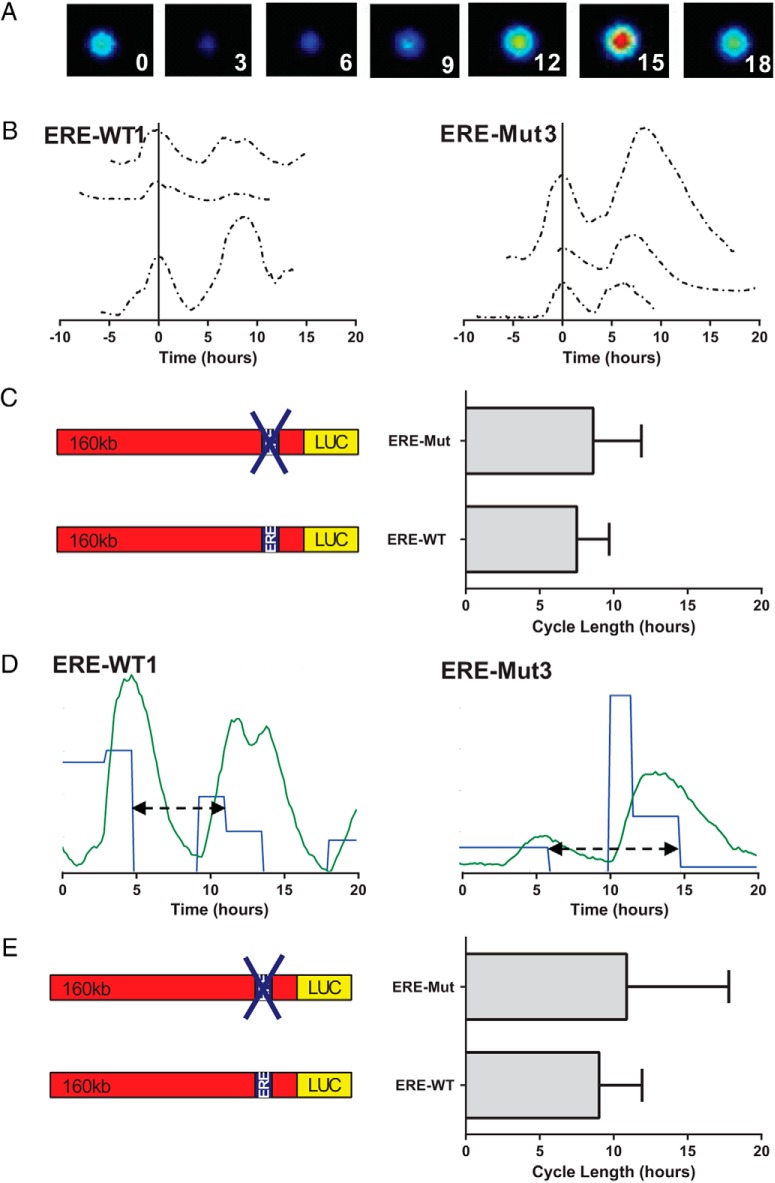
PRL promoter transcription cycle length comparison of ERE-WT1 and ERE-Mut3 BAC cell lines. Representative images from ERE-Mut3 cell over time (A). Promoter activity from ERE-WT1 and ERE-Mut3 cell lines. Each line represents the transcriptional activity from a single cell with the first peak of each cell aligned to 0 (B). Mean cycle lengths are compared between ERE-WT1 and ERE-Mut3 cell lines. Bars show SD from 13 and 15 cells, respectively, from 3 experiments per cell type (C). SSM example traces from ERE-WT1 and ERE-Mut3 cell lines showing estimated temporal switches between variable transcription rates (blue) when back calculated from observed luminescence (green). A cycle (black arrow) was determined as the duration from the first decrease in rate to the next decrease in rate having followed an intermittent increase. D, Estimated cycle length determined from SSM, compared between ERE-WT1 and ERE-Mut3 cell lines. Bars show SD of this cycle length from 10 and 21 cells, respectively, in 3 experiments per cell type (E).

The transcriptional activity of the hPRL gene is highly dynamic and pulsatile. Time lapse observations of hPRL reporter gene expression in pituitary cell lines and primary cells have found asynchronous cycles of transcription (25, 27–29), which we have proposed to be caused by phases of chromatin remodeling (30). Such patterns of gene expression may be caused by stochastic regulation of intracellular processes such as transcription factor binding, with cyclical binding leading to the ordered recruitment of cofactors to the promoters of target genes (12, 31–33). In addition binding of ER at the rPRL locus has been implicated in chromatin remodeling and also in DNA looping (17, 34). To assess whether stochastic regulation of ER binding to the −1189 ERE plays a critical role in determining patterns of hPRL promoter activity we measured hPRL promoter-directed Luc expression in single ERE-Mut3 and ERE-WT1 transfectant cells (Figure 3). Transcription cycle characteristics were examined in 3 different conditions; serum containing media (Figure 3, A–C) and serum-free media with or without estradiol (Figure 3, D–H). We found that pulsatile patterns of transcription persisted in ERE-Mut3 cells (Figure 3, A, C, and G) and that characteristics were comparable with those observed in ERE-WT1 cells (Figure 3, A, B, and E) in both serum-free and serum containing media. The mean cycle length was determined and found to be 8.9 hours (±3.3 h SD) and 7.5 hours (±2.2 h SD) for ERE-Mut and ERE-WT cells, respectively, when cultured in serum containing medium (Figure 4, A–C). These data fit well with our previous analysis of cycle length using the 160-kb hPRL promoter reporter construct where a mean cycle length of approximately 9 hours was observed (30). Additionally, mathematical analysis of single cell luminescence data using a SSM to estimate transcriptional switch timings (26), also predicted comparable cycle length times (Figure 4, D and E). These data suggest that hPRL cyclical transcription is not reliant on ER binding and supports our previous mechanistic model describing the generation of hPRL transcription cycles (30). Additionally, real-time single-cell luminescence imaging of serum-starved cells after E2 stimulation showed increased amplitude of transcription cycles in ERE-WT1 cells leading to a progressive increase over time in reporter gene expression which was not observed in ERE-Mut3 (Figure 3, E and H). This further supports the importance of the −1189 ERE in the estrogen responsiveness of the hPRL promoter.

### A biphasic hPRL transcriptional response to TNFα is abolished in ERE-Mut cell lines

**Figure 5.**
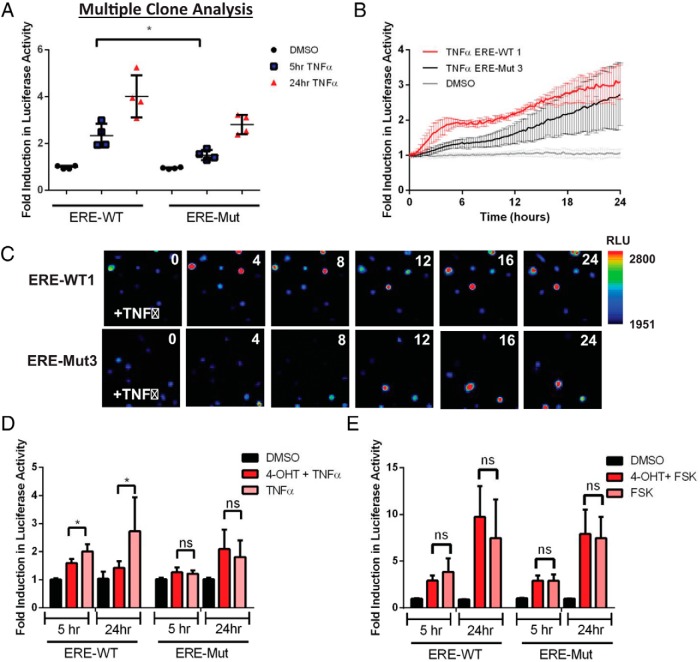
TNFα response of ERE-WT and ERE-Mut BAC-Luc cell lines. Luc activity after TNFα stimulation (10-ng/mL) of 4 individual ERE-WT and 4 ERE-Mut clones measured at 5 and 24 hours after treatment; *, *P* < .05 (A). Time series live-cell luminometry of selected clones, ERE-WT1 and ERE Mut-3 after 10-ng/mL TNFα stimulation with readings taken every 15 minutes (B). Representative luminescence images of serum-starved ERE-WT1 and ERE-Mut3 cells after 10-ng/mL TNFα treatment (C). Effect of ER inhibition on the TNFα response of ERE-WT1 and ERE-Mut3 cells using combined 4-OHT and TNFα treatment; *, *P* < .05 (D). Effect of ER inhibition on the FSK response of ERE-Mut and ERE-WT cells using 4-OHT and TNFα combined treatment (E). Results are shown as fold induction ± SD from an untreated control sample. Values represent the mean of at least 3 independent experiments, each with 3 replicates.

PRL promoter activation by TNFα is mediated by the nuclear factor-κB (NF-κB) signaling pathway and has previously been shown to have a biphasic effect over 5–24 hours using a 5-kb hPRL-Luc reporter construct (35). Mutation of the −1189 ERE in the 5-kb construct abolished the responsiveness of the hPRL promoter to TNFα treatment and our previous work indicated that combined estrogen and TNFα exerted cooperative stimulation of the hPRL gene (9). To determine the effect of this mutation on the TNFα response of the entire hPRL locus, 4 ERE-WT and 4 ERE-Mut clonal cell lines were treated with 10-ng/mL TNFα and Luc activity measured 5 and 24 hours after treatment (Figure 5A). A significant decrease in the acute transcriptional response at 5 hours was observed in the ERE-Mut cell lines compared with ERE-WT with a mean fold induction of 1.5 compared with 2.4, respectively. No significant difference was observed in the later transcriptional activation between cell lines.

Real-time live-cell luminometry of ERE-Mut3 and ERE-WT1 clones confirmed the TNFα-induced biphasic transcriptional response in the ERE-WT1 cell line with an acute increase in hPRL-directed Luc expression observed at 2–6h and a later response 12–24 hours after treatment (Figure 5B). This biphasic response was abolished in ERE-Mut3 BAC cell line (Figure 5B), in which the early response was lost but the late activation remained 24 hours after TNFα treatment. Real-time single-cell bioluminescence imaging confirmed the same pattern of hPRL transcriptional activation after TNFα treatment with loss of the acute transcriptional response observed in ERE-Mut3 cells (Figure 5C).

Cotreatment of ERE-WT cells with 4-OHT caused significant inhibition of both acute and late TNFα induced hPRL Luc activity, whereas in ERE-Mut cells no difference was observed (Figure 5D). This suggests that ER activity at the −1189 ERE plays a critical role in the acute transcriptional effect of TNFα for this gene. Control experiments showed that the hPRL FSK response was not impaired by the ERE mutation and that cotreatment with 4-OHT did not affect transcriptional activity in either cell line (Figure 5E).

### Responses of ERE-Mut and ERE-WT cell lines to well-characterized hPRL regulating stimuli

**Figure 6.**
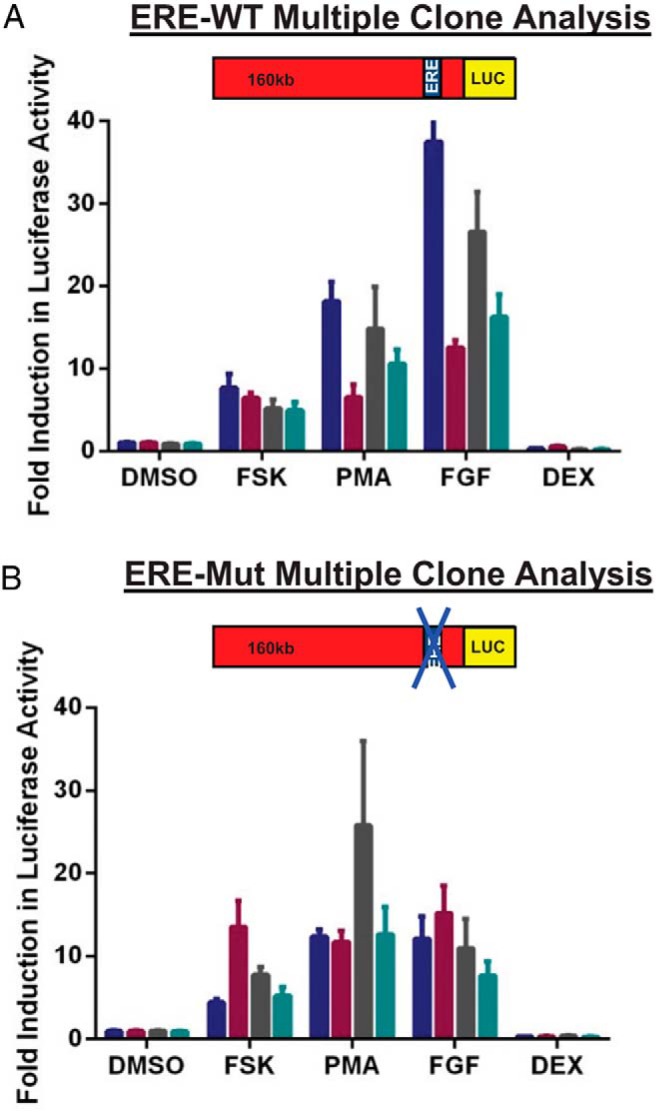
Responses of ERE-Mut and ERE-WT cell lines to well-characterized hPRL regulating stimuli. Stimulation of 4 individual serum-starved ERE-WT (A) and ERE-Mut (B) cell lines (Fsk, 5μM; FGF-2, 10 ng/mL; PMA, 10nM; Dex, 10nM). Luc activity was measured 24 hours after drug treatment. Results are shown as fold induction ± SD from an untreated control sample. Values represent the mean of at least 3 independent experiments, each with 3 replicates. Each bar represents an individual clone.

In addition to the classic genomic action of ligand activated ER, the ER can also regulate gene transcription in a number of other ways. Activation of kinase signaling cascades by growth factors and cytokines can phosphorylate and activate ER, leading to recruitment to EREs and subsequent changes in gene expression (14). To determine whether the −1189 ERE plays a role in the hPRL transcriptional response to other stimuli we compared the responses of 4 ERE-WT and 4 ERE-Mut clonal cell lines after treatment with a range of well-know hPRL regulating stimuli, including FSK, phorbol myristate acetate (PMA), FGF-2, and Dex. The transcriptional response to these stimuli was not compromised by the ERE mutation. Comparison of the mean response of clonal cell lines found no significant difference in reporter gene expression between ERE-WT and ERE-Mut cell lines (Figure 6, A and B, respectively) implying the −1189 ERE region is not involved in the hPRL transcriptional response to these stimuli.

## Discussion

Regulatory DNA elements can be situated distant from the transcription start sites of the genes they regulate and through mechanisms such as chromatin looping these distal enhancer or silencer regions can be brought into contact with proximal promoter regions to directly influence transcriptional activity (36). The use of BAC reporter constructs in molecular physiology enables the inclusion of large regions of flanking DNA and BAC recombineering allows the identification of possible long-range regulatory regions which can influence gene transcription. We have previously generated a BAC-reporter gene construct expressing Luc under the control of 163 kb of the hPRL locus (hPRL-BAC-Luc). Using the hPRL BAC-reporter gene construct we have studied hPRL gene expression under different physiological conditions in both stably transfected GH3 pituitary cell lines and primary pituitary cells from transgenic rats (1, 2, 27, 37). Here, we have further engineered the hPRL-BAC construct to determine the potential contribution of extended genomic regions to the estrogen responsiveness of the hPRL promoter, whether the −1189 ERE is crucial, and its function depends on genomic context and other enhancer elements or there are additional more distal ERE's involved in the hPRL E2 response. Using a GalK strategy (24), we have mutated the previously defined variant −1189 ERE in the hPRL-BAC-Luc construct and compared the response of stable cell lines expressing these constructs (ERE-WT and ERE-Mut) to a range of well-characterized hPRL regulating stimuli. Mutation of the −1189 ERE dramatically altered the promoter's responsiveness not only to E2 but also to TNFα. Unexpectedly, disruption of this crucial DNA element had no effect on the characteristic pulsatile cycles of hPRL transcription, which were observed in both ERE-WT and ERE-Mut transfectant cells.

Estrogen is an important physiological regulator of PRL expression. It is known to increase lactotroph cell growth and activate PRL gene expression both in vivo using rodent models and in vitro in rat pituitary cell lines and dispersed primary pituitary cell cultures (9, 11, 19, 38, 39). Transgenic mice carrying a BAC transgene containing the entire hPRL locus exhibit tissue expression of hPRL, which in pituitary lactotrophs is responsive both in vivo and in vitro to E2 treatment (11). Continuous E2 treatment for 14 days via an implanted pellet led to significantly increased pituitary weight and hPRL gene expression, and in vitro E2 treatment of primary pituitary cell cultures from these animals led to increased hPRL protein (11). Estrogen-mediated regulation of hPRL transcription is facilitated by binding of ERα to the −1189 ERE and induces modest reporter gene activation in hPRL-Luc stable transfectant pituitary cells (5-kb hPRL-Luc) (9). However, cells expressing Luc under the control of a 160-kb fragment of the hPRL gene locus display a significantly greater transcriptional response to E2 than the 5-kb promoter construct (1). This implied that the larger genomic fragment may contain additional sites involved in estrogen-mediated activation of the hPRL gene, either additional more distant EREs or other upstream sequences required for the full responsiveness of the known proximal ERE. The promoter regions of E2 responsive genes can contain numerous EREs (40, 41) and transcriptional synergism after ER binding to multiple EREs has been reported (41–43). Additionally, chromosome-wide identification of EREs in human and mouse genomes found that EREs are frequently situated far distant from the genes they regulate (21, 44) with only 5% of the mapped ERE-binding sites in MCF-7 cells found within 5-kb region of the transcription start site (45). The involvement of upstream sequences in estrogen-induced transcriptional regulation of the rPRL promoter has also been reported with chromatin looping mediated by the −1575 ERE suggested as a mechanism of E2-induced transcription at the rPRL locus (34).

Analysis of the hPRL-BAC sequence for potential ERE motifs was performed using Dragon ERE finder version 3 (46). This identified a number of potential long-range EREs with 4 sites being within 1 kb of a forkhead box protein A1 (FoxA1)-binding site (data not shown). This “pioneer” factor is suggested to be required for long-range gene activation, binding close to EREs to facilitate ER binding (21). In addition, a functional ERE has been identified in the hPRL distal promoter (approximately −6950 kb from the pituitary transcription start site), and shown using promoter reporter constructs in T47D human breast cancer cells to induce hPRL promoter activity in response to E2 (10). However, it is likely from our data that any more distal, putative EREs have very little functional effect in the estrogen-mediated activation of the hPRL promoter. Mutation and subsequent loss of ER binding to just the single −1189 ERE within the 160-kb hPRL genomic fragment resulted in complete loss of sensitivity to E2 in 2 of the ERE-Mut transfectant clones screened, and minimal induction in a further 3 lines, suggesting that this relatively proximal ERE is the dominant response element even in the context of the very large genomic fragment. Furthermore, full estrogen-mediated activation of the hPRL promoter in T47D cells did not involve the −1189 proximal ERE but required the cooperative action of ERs on both ERE and AP-1 sites in the distal promoter (10), suggesting different mechanisms involved in the E2-mediated regulation of the hPRL promoter in extrapituitary and pituitary cells. It is possible that intrachromosomal interactions between this crucial −1189 ERE and an upstream enhancer region may be responsible for the enhanced E2-mediated transcriptional activation observed in cells transfected with the 160-kb hPRL BAC compared with the 5-kb hPRL construct. This hypothesis would require further work to test in detail, application of circularized chromosome conformation capture (4C) assays may identify unknown DNA regions interacting with the −1189 ERE.

Interactions between the ER and NF-κB signaling pathways have been widely reported in multiple systems with both positive and negative cross talk effects described (47–50). Cooperative binding of both transcription factors at adjacent response elements in the promoter region of the ABCG2 gene stabilizes their interaction after combined TNFα and E2 treatment, leading to enhanced expression of this estrogen responsive gene (48). Additionally, TNFα can activate ER signaling in human endometrial epithelial cells transfected with an ERE-Luc reporter, with a significant increase ERE transcriptional activity observed after TNFα treatment in the absence of ligand (16). However, TNFα had no effect on ERE transcriptional activity in GH3 rat pituitary cells transfected with a consensus ERE-Luc reporter (9), suggesting cell type and/or species-specific differences in ER and NF-κB signaling cross talk.

Using a 5-kb hPRL-Luc reporter, we have previously shown that the presence of the −1189 ERE region is essential for TNFα-mediated activation of the hPRL promoter, measured 24 hours after TNFα treatment (9). In the present study, we show that mutation of this ERE in the 160-kb hPRL BAC results in loss of the acute TNFα transcriptional response, but no significant effect on the later, 12- to 24-hour transcriptional activation. Cotreatment of ERE-WT cells with 4-OHT significantly reduces the acute hPRL transcriptional TNFα response to levels comparable with ERE-Mut cells, indicating ERα activity at the −1189 ERE is essential for the ability of TNFα to acutely enhance hPRL gene expression. The absence of any difference in the later, more delayed TNFα transcriptional response between ERE-Mut and ERE-WT hPRL BAC cell lines suggests that this slower response is likely to be mediated by upstream regulatory regions contained in the extended genomic sequence, not present in the 5-kb hPRL promoter construct.

The hPRL promoter region contains multiple response elements, binding transcription factors involved in both positive and negative regulation of the gene. These include binding sites for pituitary-specific transcription factor 1 (Pit1) (51, 52), AP-1 (10, 53), E26 transformation-specific (ETS) domain factors (ETS-1) (54) in addition to the −1189 ERE. The hPRL −1189 ERE is a putative critical response element which could have contributed to the transcriptional response to other hPRL regulating stimuli. However, treatment of multiple ERE-WT and ERE-Mut clonal cell lines with FSK, FGF, PMA, and DEX revealed that there was no significant difference in the transcriptional response to these stimuli between the 2 cell lines after treatment for 24 hours (Figure 6, A and B). This indicates that the −1189 ERE is not involved in the transcriptional response to these stimuli.

Cyclical, dynamic patterns of hPRL gene expression over time have been shown using real-time bioluminescence and fluorescence imaging of hPRL-BAC (1, 27) and hPRL-5-kb (25, 28–30) reporter constructs to visualize hPRL promoter directed reporter gene activity in individual living cells (30). Additionally, pulsatile patterns of hPRL gene expression were observed in both adenovirally transduced GH3 and primary Syrian hamster pituitary cells, using an adenoviral vector expressing Luc regulated by the hPRL promoter. Dynamic hPRL transcriptional activity was observed in both the clonal cell lines and in primary cells (28). In all of the model systems that have been studied, heterogeneous, dynamic cycles of hPRL transcription have been observed, showing that the responses are not affected by construct type, promoter length or site of integration. This phenomenon appears to be a fundamental property of the transcription process with transcriptional bursting observed from numerous mammalian genes (55).

Analysis of ERα binding to the proximal promoter regions of several estrogen responsive genes has shown cyclical recruitment after E2 treatment with subsequent ordered recruitment of cofactors to the target genes (12, 17, 20). This mechanism has been proposed to be a general property of ERα regulated genes. Given the cyclical nature of hPRL gene transcriptional activity and its estrogen responsiveness we hypothesized that cyclical binding of ERα to the degenerate ERE may play a critical role in generating the cycles or bursts of transcriptional activity. Surprisingly real-time bioluminescence imaging of single clonal ERE-Mut transfectant cells showed that oscillatory patterns of transcription persisted, with cycle lengths comparable with those observed in ERE-WT clonal cell lines (Figure 4). This suggests that cyclical fluctuations of promoter activity are a fundamental feature of the transcription complex, at least at this locus, and that they are not dependent upon ER binding or activity. This supports our previous mechanistic model of hPRL transcription, proposing that chromatin remodeling processes, rather than individual endocrine signals, generate on and off cycles of transcription (30).

In summary, we have used BAC recombineering to identify the critical role of a single, relatively proximal ERE in controlling PRL transcription. This ERE controls not only the response to estrogen but also the transcriptional response to TNFα. Crucially, we have used real-time bioluminescence imaging of transfectant cells to reveal that cycles of transcriptional activation in individual living cells are maintained despite the disruption of this key ERE. This indicates that transcriptional cycles are independent of ER binding and activation and are not directly controlled by hormonal signaling.

## Additional material

Supplementary data supplied by authors.
