# Supplementary material for: Role of Estrogen Response Element in the Human Prolactin Gene: Transcriptional Response and Timing
Source: Mol Endocrinol. 2015 Dec 21;30(2):189–200. doi: 10.1210/me.2015-1186 (PMC4792233; doi:10.1210/me.2015-1186)

Supplemental Figure 1

Confirmation of -1189 ERE mutation

Mutation of the WT ERE results in the formation of an EcoNI restriction site. Eight colonies were screened by amplification across the target region and subsequent digest of the PCR reaction with EcoNI. WT band is 332bp, whereas in modified constructs (including plasmid control from [9]) digestion resulted in two bands of 191 and 141bp. Sequencing confirmed these clones were correctly mutated (a) Southern blots and Pulsed Field Gel electrophoresis were used to confirm the overall BAC structure was maintained of the 8 clones tested. Probes targeting 5' region of the BAC (probe I), the central region (probe II) and the 3' region (probe III) confirmed the presence of these regions in all BACs, as in the WT BAC. (b)(i) SalI and NotI digestion and PFGE confirms BAC overall structure is maintained (b)(ii).

a.

EcoN1 Screening

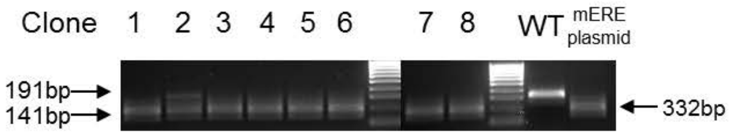

b. (i)

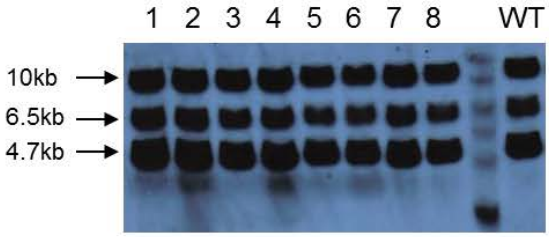

Southern Probe	1st digest position	2nd digest position	Fragment Size (bp)
I	14706	21276	6570
II	125796	127626	4700
III	152109	162170	10061

(ii)

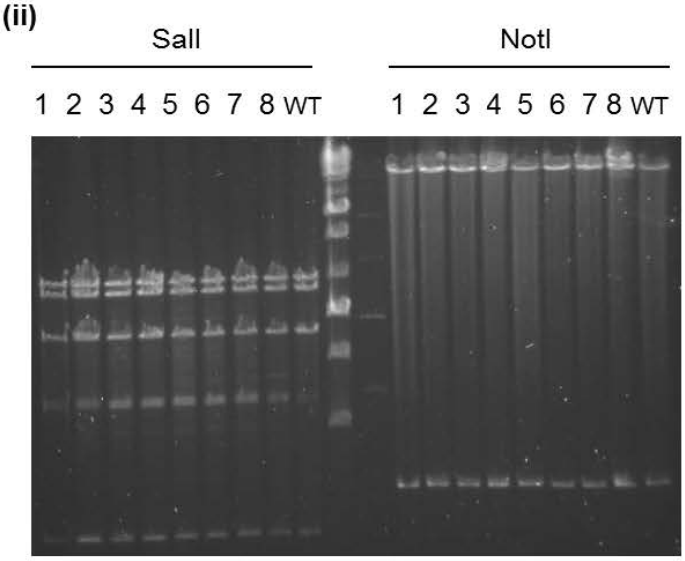

Supplement: Supplementary file 1 [file me-15-1186.pdf]
